# A Rare Cause of Bilateral Corneal Ulcers: Vitamin A Deficiency in the Setting of Chronic Alcoholism

**DOI:** 10.7759/cureus.7991

**Published:** 2020-05-06

**Authors:** Raman J Sohal, Thu Thu Aung, Sandeep Sohal, Abha Harish

**Affiliations:** 1 Internal Medicine, State University of New York (SUNY) Upstate Medical University, Syracuse, USA; 2 Internal Medicine, The Brooklyn Hospital Center, Brooklyn, USA

**Keywords:** bilateral corneal ulcers, vitamin a deficiency, alcoholism, liver cirrhosis, nutritional deficiency

## Abstract

Vitamin A deficiency is rarely encountered in the western world. When encountered, vitamin A deficiency is seen as a component of the malabsorption spectrum of disease. Given the infrequency of nutritional deficits in the developed world, vitamin A-associated ophthalmologic disease is rarely encountered. We report a case of a 56-year-old male with severe vitamin A deficiency in the setting of alcoholic liver cirrhosis. This case emphasizes two important points. First, it considers vitamin A deficiency as a cause of corneal ulceration in patients with chronic alcoholism. Second, it raises awareness of hepatotoxicity that can result after the supplementation of vitamin A in patients with chronic alcoholism. Although an uncommon diagnosis, it should be considered when other causes, such as infectious and autoimmune conditions, are ruled out.

## Introduction

Vitamin deficiency is not a commonly encountered cause of non-healing corneal ulcers. However, in cirrhotic patients when other differentials, such as infectious and autoimmune etiologies, have been excluded, vitamin A deficiency should be considered. In patients with liver cirrhosis, the deficiency stems from both decreased gastrointestinal absorption as well as decreased oral intake. A common physical presentation is Bitot spots, which is keratinous debris that accumulates on the surface of the cornea [1]. Initially, there is a combination of both corneal and conjunctival xerosis that is followed by Bitot spot deposition and then ends with keratoconjunctivitis. It may become so extensive as to have corneal necrosis.

Vitamin A is necessary for epithelialization and mucin production. It is the leading cause of blindness worldwide. Corneal lesions can vary from punctate to complete perforation. Hypovitaminosis A produces keratitis that is refractory to typical treatment with lubricants and topical eye drops [1-2]. Frequently, these patients will have had a significant workup for multiple autoimmune conditions, such as Sjogren syndrome, which have come back negative.

In developed countries, the main diseases associated with vitamin A deficiency are those of malabsorption syndromes such as cystic fibrosis, ulcerative colitis, Crohn's disease, bariatric surgery, short bowel syndrome, celiac disease, and, less commonly mentioned, liver disease [1,3]. The liver plays a large role in the catabolism of vitamin A. Vitamin A is fat-soluble and absorbed through the lymphatic channels. There is reduced hepatic vitamin storage and release by binding proteins. These patients are also deficient in the conversion of beta carotene into retinol (the active form of vitamin A) [4-5].

Alcohol adversely affects vitamin A metabolism, as retinol and alcohol share a common oxidative and non-oxidative pathway. Both are substrates of alcohol and retinol dehydrogenase. Chronic alcoholism leads to a decreased hepatic concentration of retinol and retinoids (retinoic acid and retinyl esters). These decreased stores are independent of decreased oral intake and absorption. Research on rat and baboon models have shown that alcohol works to inhibit retinoic acid formation [2,4]. Alcohol also increases the extrahepatic metabolism of retinoic acid [4,6]. In our case, we used a lower dose than recommended in order to reduce the possible effects of the toxicity given the degree of ongoing liver disease.

Prokera® (Bio-Tissue, Inc., Miami, FL) is a non-adhesive amniotic membrane that is transplanted onto the site of corneal ulceration to promote healing. It builds a layer between the ulcer site and the eyelid, thereby providing protection in addition to intrinsic anti-inflammatory, wound healing, and anti-scarring properties [6]. It also helps to increase stem cell proliferation at the corneoscleral limbus. It can be removed after a maximum of 29 days but it often self-dissolves after three to seven days [7-8]. The non-dissolving conformer is then removed. This was done in our patient.

## Case presentation

We present a case of a 56-year-old male who initially presented obtunded to an outside hospital with a Glasgow Coma Score (GCS) of 15 due to ethanol intoxication. Upon improvement in his mental status, the patient complained of intense pain and purulent drainage from both his eyes for a few days. He was noted to have hyphema and hypopyon with obstruction of the iris and pupil of the left eye and thus was transferred to an academic referral center that had an inpatient ophthalmology consult service. He complained of being unable to open either eye without significant discomfort but did deny changes in visual acuity. On arrival, the patient was also found to have acute alcoholic hepatitis with ascites, jaundice, tender hepatomegaly, significant transaminitis, leukocytosis, hypoalbuminemia, and elevated international normalized ratio (INR). No paracentesis was done.

**Figure 1:**
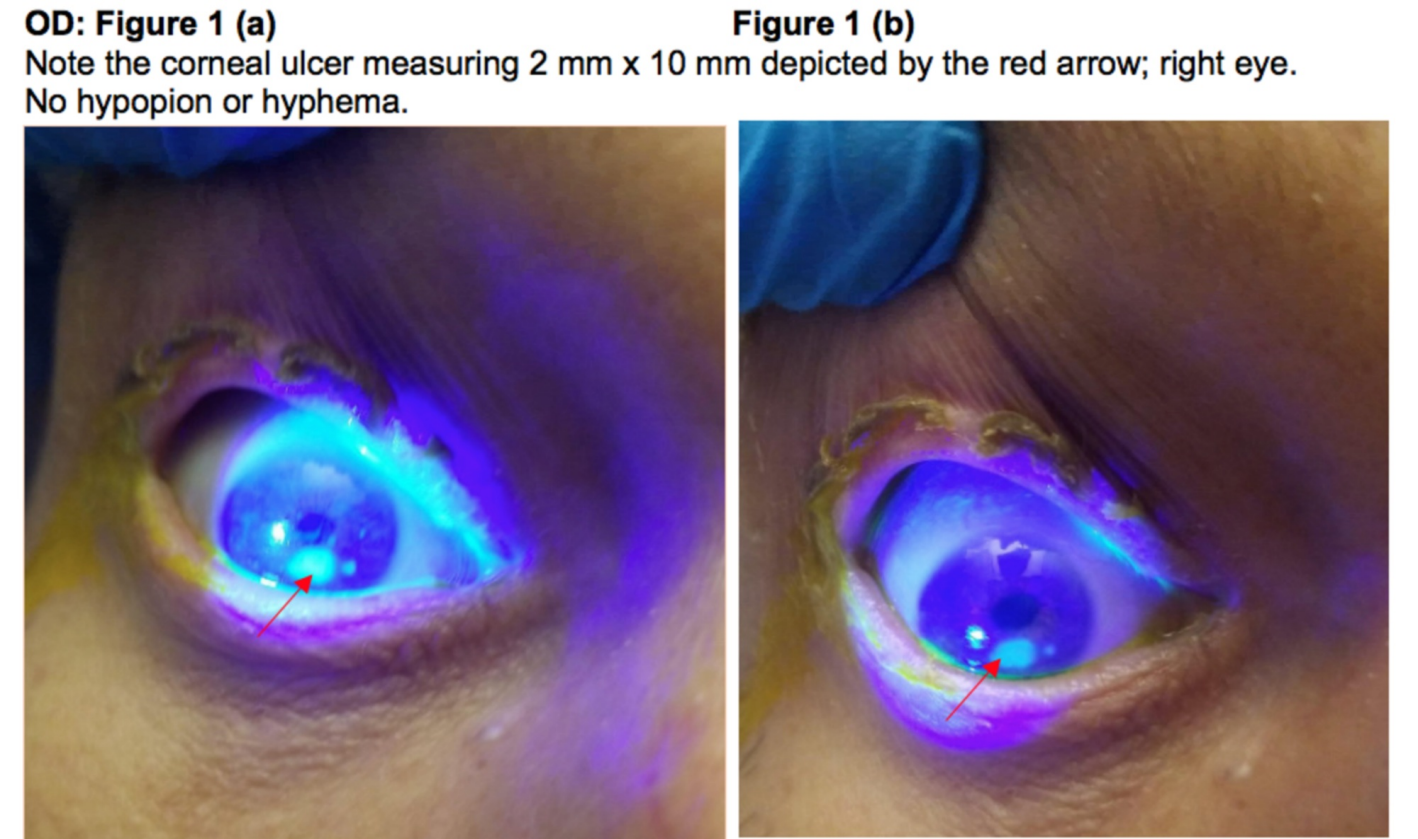
Right eye OD: oculus dexter (right eye)

**Figure 2:**
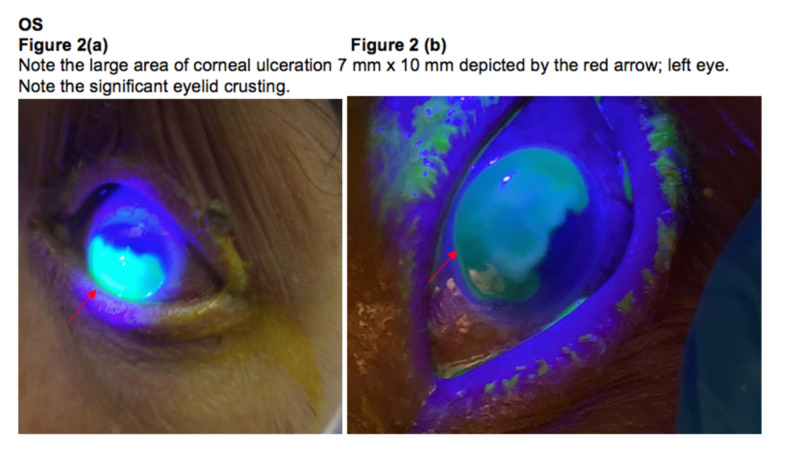
Left eye OS: oculus sinister (left eye)

The patient noted a progressive visual decline since the past one month. Cultures from eye drainage grew 2+ Corynebacterium macginleyi that was pan-sensitive from both eyes. On exam, the right eye was more sensitive to touch than the left. He was started on fortified vancomycin and tobramycin oculus uterque (OU; both eyes). Initially, the regimen was to administer the antibiotics every five minutes for 15 minutes (three rounds), then switch to every one hour alternating so the patient was getting an eye drop every 30 minutes. Atropine drops were also started twice daily OU. B-scan ultrasonography was performed on Day 1, which showed no vitritis in the left eye. The patient underwent PROKERA Plus (Bio-Tissue, Inc., Miami, FL) implant in the left eye on Day 2 (48 hours after antibiotic therapy), which was removed on Day 6. PROKERA was then replaced bilaterally on Day 6 and then removed on Day 10. Subconjunctival Vigamox was administered bilaterally on Days 3 and 6. Epithelial defects remained nearly unchanged in the right eye, now with slight thinning that was seen but stable. The left eye continued to epithelialize slowly. Given the pan-sensitivity of the culture results, doxycycline 100 mg twice daily was started for two weeks. During the first few days of the physical exam, in the left eye, there was 2+ injection, a subconjunctival hemorrhage located near the ring of PROKERA, with trace purulent discharge of the left eye. The ulcer was approximately 7 mm x 10 mm ring-like infiltrate with heaped-up edges especially in the superior central zone with roughened, hazy epithelium. There was a regressing, U-shaped, arcuate-like epithelial defect surrounding the ring, sparing the superior cornea. There was minimal thinning at best. The right eye had a 2 mm epithelial defect with stromal haze with 10% thinning at best. At the time of this exam, Prokera was present in both eyes. Over the course of 10 days, the ulcers showed some improvement; however, there were still non-healing epithelial defects despite several rounds of PROKERA, which brought up the possibility of requiring serial ocular/corneal surgeries (such as a lateral tarsorrhaphy) for the maximal attempt at visual rehabilitation once the infection had cleared. Please see Figure 1 and Figure 2, which show the right and left eyes, respectively, following the application of the fluorescein dye and then observation under blue light.

Late evening of Day 10, the patient developed new-onset abdominal pain and melena. Esophagogastroduodenoscopy (EGD) was performed on Day 11, which showed non-bleeding duodenal ulcers and gastric ulcers. The following day, the patient had acute blood loss anemia with a drop in hemoglobin, along with hemodynamic instability. Computed tomography (CT) angiogram of the abdomen and pelvis showed no active bleed. The patient was given Kcentra from elevated INR. Interventional radiology was consulted to perform a visceral arteriogram of the celiac, superior, and inferior mesenteric area with no active bleed identified again. Later that day, an abdominal radiograph was performed due to worsening pain, which showed free air under the diaphragm. This was suggestive of visceral perforation. The patient was taken for emergent exploratory laparotomy and found to have a perforated duodenal ulcer after which he was taken to the surgical intensive care unit (ICU) for increasing pressor requirements. He developed a recurrent upper gastrointestinal (GI) bleed on Day 13, this time requiring a massive blood transfusion protocol. A repeat EGD was performed, which showed a clot in the duodenum without active source. He was taken for prophylactic coil embolization of the gastroduodenal artery by interventional radiology. The patient was extubated on Day 15; however, shortly after, he developed increasing shortness of breath and acute hypoxic respiratory failure requiring reintubation. He subsequently developed increased bloody output from the nasogastric tube, as well as melanotic stools with an associated drop in hemoglobin/hematocrit (H/H). A repeat computed tomography angiography abdomen and pelvis (CTA A/P) on Day 17 revealed active bleeding into the second portion of the duodenum. He was taken to interventional radiology (IR) for a visceral angiogram, which suggested active bleeding from a branch of the inferior pancreaticoduodenal artery (IPDA). He underwent selective distal IPDA branch embolization. The patient's poor prognosis from his liver failure was discussed with family and they decided to proceed with comfort measures only. He was pronounced deceased subsequently.

## Discussion

Ophthalmologic complications are not frequently seen as a consequence of liver cirrhosis. When present, vitamin A deficiency is a rare reason for keratomalacia. Such deficiency is rare in the developed world and typically seen in patients with malabsorption syndromes; however, this case reports on the presence of alcoholic cirrhosis as the cause of vitamin A deficiency.

Our patient was started on high dose vitamin A supplementation at 50,000U per day for three days intramuscularly and then weekly. The initial level was 3.5 ug/dL on presentation and increased only slightly to 3.7 ug/dL (Ref. range of 20.1-62.0 ug/dL) after treatment. Patients with a level of less than 10 ug/dL are considered severely deficient. A zinc level was checked as well, which was low: 52 (ref. range 56-134 ug/dL).

The typical starting dose of vitamin A is 200,000 U PO on the day of diagnosis followed by 200,000U the following day and then a third final dose one week later [4]. For severe vitamin deficiency, it is started at 100,000 U as an intramuscular injection with the same schedule timing. In our case, the decision to start at 50,000 U intramuscularly was made. There are no formal recommendations for the treatment of severe vitamin A deficiency. In our patient, a more conservative approach was chosen, as vitamin A at high levels along with concurrent ethanol use can potentiate hepatotoxic effects of vitamin A when combined, thereby narrowing the therapeutic index of vitamin A in patients with chronic alcoholism. In our patient, 50,000 U IM vitamin A was administered as opposed to the recommended dose of 100,000 U IM. Unfortunately, our patient passed away from other causes, and we were unable to track the progression of the corneal ulceration. Despite dose reduction, there was worsening hepatic synthetic function following the administration of the vitamin A intramuscular shots. Total bilirubin was 3.1 on Day 1 of administration and this increased to 4.6 on Day 2 followed by 5 mg/dL on Day 3 of administration. Ammonia increased to 68 on Day 3. On Day 2, the patient’s INR was 4.42 after which he was given Kcentra (Prothrombin complex concentrate including factors II, VII, IX, X; factor IV unactivated). To be noted, the patient was not on anticoagulation, however, in an effort to reduce the bleeding risk, this was given.

## Conclusions

In summary, vitamin A deficiency results in progressive symptoms from nyctalopia, to corneal degeneration, to complete retinal perforation. It functions as an essential transcription for hundreds of genes involved in epithelial turnover, mucin production, and for the functioning of rods and cones. Deficiency of this vitamin is uncommon in the Western world and if it occurs, it usually does so in the setting of a malabsorption disorder or bariatric or bowel resection surgery. Alcohol and retinol compete as substrates for a common pathway of metabolism. Often, this leads to the depletion of intra-hepatic retinol stores and the increased mobilization of extra-hepatic stores. This report emphasizes the incidence of severe vitamin A deficiency in the chronic alcoholism population. Vitamin A deficiency can present as bilateral corneal ulcerations, and it should be considered a differential diagnosis of keratitis especially if other causes as infection and autoimmune disease have been ruled out. This article emphasizes the delivery of vitamin A with caution, as there is the possibility of producing worsening hepatotoxicity.
